# Uncovering the Uncommon: An 81-Year-Old Woman With Elevated Carcinoembryonic Antigen (CEA) but Normal Calcitonin Levels Diagnosed With Medullary Thyroid Carcinoma

**DOI:** 10.7759/cureus.40290

**Published:** 2023-06-12

**Authors:** Janta Ukrani, Martin Barnes, Aqsa Ashraf, Gregory Haggerty, Noshir Dacosta

**Affiliations:** 1 Internal Medicine, Mather Hospital Northwell Health, Port Jefferson, USA; 2 Hematology and Oncology, Mather Hospital Northwell Health, Port Jefferson, USA; 3 Graduate Medical Education, Mather Hospital Northwell Health, Port Jefferson, USA; 4 Hematology and Medical Oncology, New York Cancer and Blood, New York, USA

**Keywords:** immunohistochemistry staining, thyroid nodules, elevated carcinoembryonic antigen (cea), normal calcitonin, medullary thyroid carcinoma

## Abstract

Medullary thyroid carcinoma (MTC) is a rare neuroendocrine tumor. However, non-elevated calcitonin levels have been reported in the literature. We present the case of an 81-year-old woman with chronic elevations in carcinoembryonic antigen (CEA) levels for the past 15 years, despite normal calcitonin levels, who was ultimately diagnosed with MTC. The patient had a remote history of breast cancer and presented with symptoms of unintentional weight loss, fatigue, and joint pain. A positron emission tomography (PET) scan revealed low fluorodeoxyglucose (FDG) uptake in partially calcified thyroid nodules, and fine needle aspiration cytology was consistent with medullary carcinoma. The patient underwent total thyroidectomy, with pathology revealing a pT1aN0M0 medullary thyroid microcarcinoma with negative margins. After thyroidectomy, CEA levels decreased to within the normal range, and calcitonin levels remained normal. This case highlights the importance of considering MTC in patients with unexplained chronic elevations in CEA levels, even with normal calcitonin levels.

## Introduction

Medullary thyroid carcinoma (MTC) is a rare neuroendocrine tumor that originates from the parafollicular cells of the thyroid gland, representing 1%-10% of all thyroid cancers with a 10-year survival rate between 69-89% [1, 2]. MTC typically presents with a palpable neck mass or cervical lymphadenopathy and is associated with elevated levels of calcitonin [3]. Calcitonin is a 32-amino acid polypeptide hormone and is considered a diagnostic marker with a sensitivity of around 98%-99% [4]. Parafollicular cells secrete calcitonin, carcinoembryonic antigen (CEA), neuron-specific enolase, and chromogranin A, and their serum concentrations generally reflect tumor burden [1]. MTC can be classified into sporadic and hereditary forms. The sporadic form occurs without a family history and accounts for approximately 75% of cases, while the hereditary form is associated with an autosomal dominant inheritance pattern and can be further divided into isolated familial MTC and multiple endocrine neoplasia type 2 (MEN 2) [5]. Mutations in the RET (REarranged during Transfection) proto-oncogene are the main molecular etiology of hereditary MTC [5].

MTC with non-elevated calcitonin levels has been reported in the literature, although it is relatively rare. According to the literature, approximately 5% of MTC cases have normal serum calcitonin levels at the time of diagnosis [6]. Our case involves an 81-year-old woman with a remote history of breast cancer and no evidence of disease who presented with documented, unexplained elevations of carcinoembryonic antigen (CEA) levels that persisted for 15 years despite normal calcitonin levels. This ultimately led to the diagnosis of medullary thyroid cancer (MTC).

## Case presentation

An 81-year-old woman with a medical history of stage IA hormone receptor (HR) positive, human epidermal growth factor receptor-2 (HER-2) negative breast cancer presented to the outpatient clinic with an elevated carcinoembryonic antigen level (CEA), which had remained elevated for the past 15 years. She was diagnosed with breast cancer in 2007 and underwent a partial mastectomy, following which she was started on adjuvant endocrine therapy with exemestane, an aromatase inhibitor, for 10 years. The patient had a past medical history of a pulmonary embolism in 2019, stroke with no residual focal deficits in 2017, osteoporosis, hyperlipidemia, and diabetes mellitus type II, all well controlled. She never smoked cigarettes, and her family history was significant for ovarian cancer in her mother and grandmother and breast cancer in her maternal aunt. In the past couple of years, she has had complaints of unintentional weight loss of around 50 pounds, fatigue, and joint pain. The patient denied symptoms of hoarseness, tachycardia, dyspnea, dysphagia, nasal congestion, shortness of breath, and cough.

**Figure 1:**
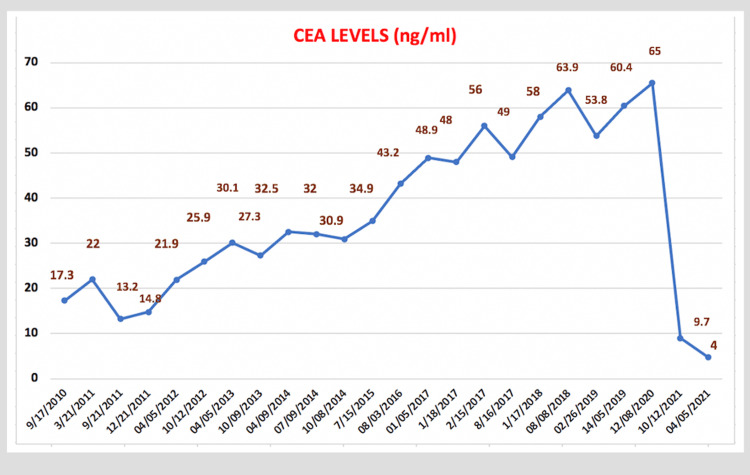
Graph showing changes in CEA from the first visit to post-thyroidectomy

Her physical examination was significant for a healed right lumpectomy scar, and no thyroid nodules or lymph nodes were palpated. The exam was negative for a wheeze, stridor, or shortness of breath. The patient had an unremarkable cardiovascular, abdominal, and neurological exam. Breast panel and tumor markers including CA-125, CA-27.29, and CA 19.9 showed normal values. However, the patient had chronic, progressively raised CEA levels ranging from 17 to 65.5 ng/ml (normal value 0-5 ng/ml) from 2010 to 2021 (Figure 1).

**Figure 2:**
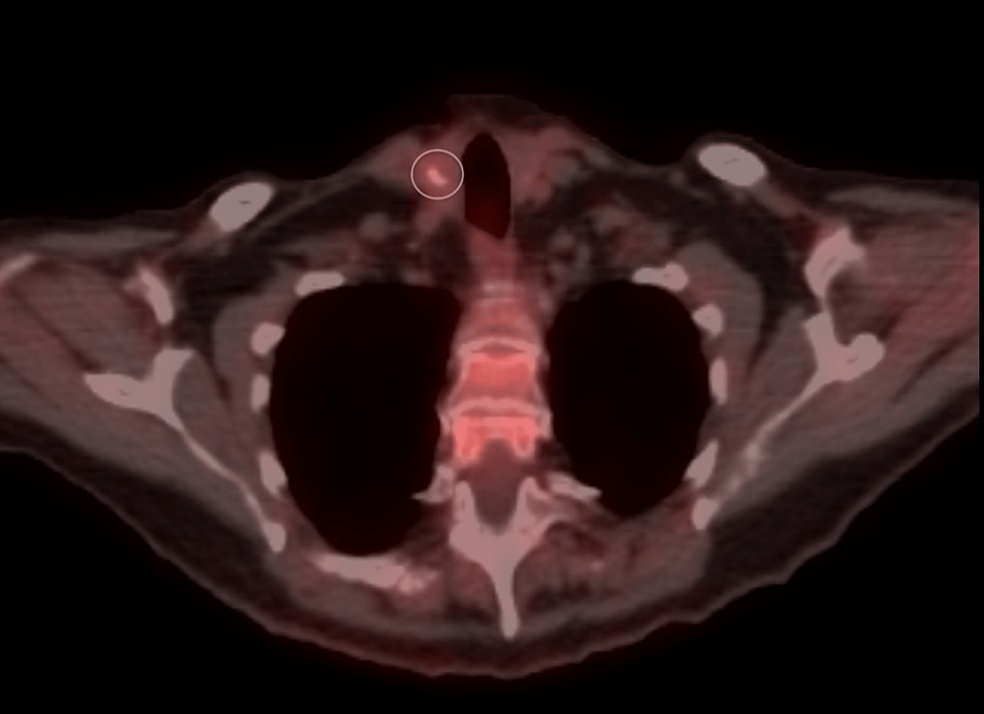
The PET-CT scan shows a partially calcified right thyroid nodule

In 2020, the patient underwent a computed tomography (CT) scan of the abdomen, chest, and pelvis, which showed no evidence of malignancy. Additionally, a mammogram showed benign fibrocystic changes. The patient underwent a positron emission tomography (PET) scan a year later, which showed low levels of F-18-deoxyglucose (FDG) uptake within partially calcified right thyroid lobe nodules with a standardized uptake value (SUV) of 2.3 (Figure 2). Subsequently, an ultrasound performed one month later showed several hypoechoic right thyroid nodules up to 1.1 cm with coarse calcification and no suspicious central lymphadenopathy.

**Figure 3:**
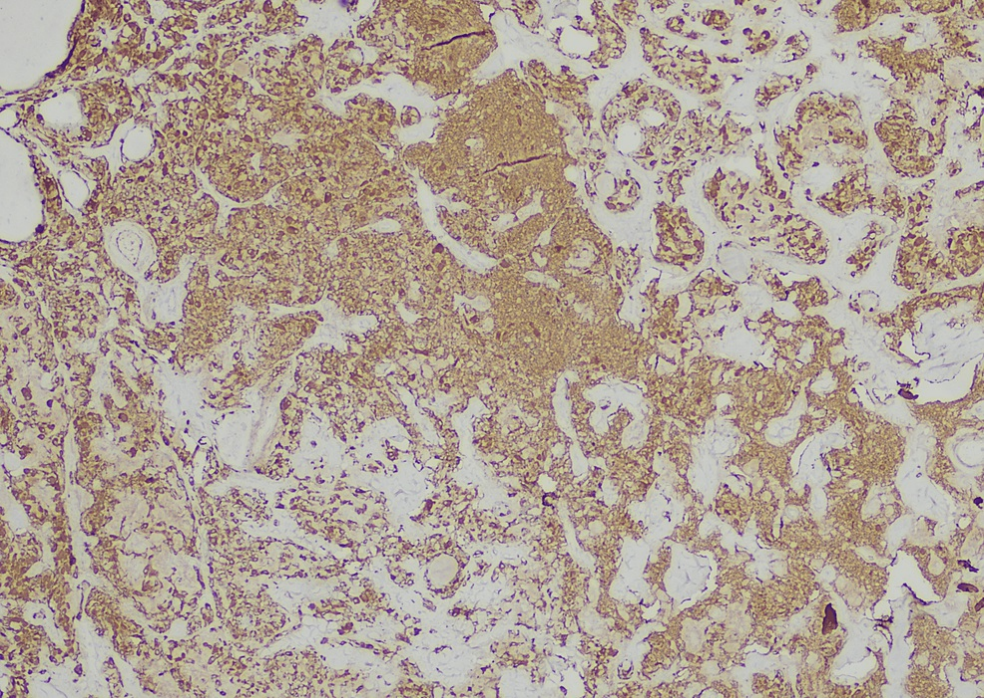
Definitive pathology of the thyroid tissue with microscopy and immunochemistry evaluation: The nests of neoplastic cells were separated by thick septa of fibrous tissue (hematoxylin and eosin, original magnification, x 100). Neoplastic cells are synaptophysin and chromogranin-positive (neuroendocrine markers are positive in medullary thyroid carcinomas). Thyroglobulin is negative in the tumor but positive in the surrounding non-neoplastic thyroid tissue.

**Figure 4:**
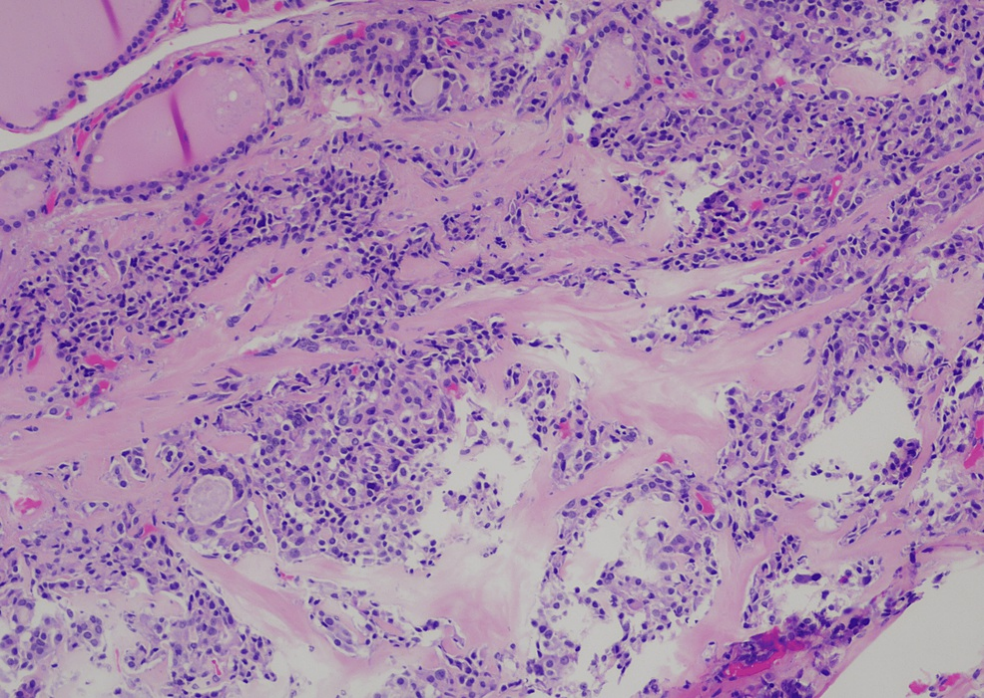
Definitive pathology of the thyroid tissue with microscopy and immunochemistry evaluation: The nests of neoplastic cells (hematoxylin and eosin, original magnification, x 100)

A fine needle aspiration was performed, and the cytological result was consistent with medullary carcinoma. Peripheral blood work showed normal calcitonin levels of < 2 ng/ml. Results of thyroid function tests, anti-thyroid antibodies, plasma catecholamine, fractionated metanephrines, norepinephrine, dopamine, and normetanephrine were all within the normal range. A preoperative CT of the chest and abdomen revealed no other extrathyroidal lesions, and the patient underwent a total thyroidectomy. Surgical pathology revealed a medullary thyroid microcarcinoma measuring 0.7 cm in the right lobe, with three benign lymph nodes and margins negative for the tumor. Pathologic staging of the right superior lobe tumor was consistent with pT1aN0M0 (Figures 3-4).

Immunohistochemistry staining was positive for CAM 5.2, synaptophysin, chromogranin, and thyroid transcription factor-1 (TTF-1) and negative for calcitonin. After thyroidectomy, CEA levels dropped from 65.5 to 9 and eventually to 4 ng/ml (Figure 1), with sustained normal-range calcitonin levels of < 2 ng/ml. On genetic analysis, the patient tested negative for multiple endocrine neoplasia type 2 (MEN 2) and mutations of the RET proto-oncogene. She was suggested for RET genetic testing and calcitonin levels for screening in her children.

## Discussion

This case report describes a unique presentation of medullary thyroid cancer (MTC) with progressively elevated levels of carcinoembryonic antigen (CEA) leading to the diagnosis. While elevated calcitonin levels are a useful diagnostic biomarker for medullary thyroid carcinoma (MTC), some patients with poorly differentiated MTC or metastasis may not have elevated levels of calcitonin. In these cases, measuring serum levels of carcinoembryonic antigen (CEA) can be a helpful diagnostic tool [6,7]. CEA is a glycoprotein that is normally produced during fetal development and is present in trace amounts in healthy adults. However, elevated levels of CEA can indicate the presence of certain types of cancer, including MTC. In fact, measuring CEA levels is considered a useful diagnostic tool for MTC, particularly in cases where calcitonin levels are not elevated [6-8]

It's worth noting that while CEA can be a helpful diagnostic biomarker for MTC, it is not specific to this type of cancer and can also be elevated in other types of tumors. Therefore, CEA measurements should be used in conjunction with other diagnostic tests and clinical assessments to make an accurate diagnosis and determine the most appropriate treatment plan [6,8]. The patient in this case had elevated CEA levels early on, which remained consistently elevated throughout follow-up appointments. This justified ordering a PET scan and led to the serendipitous discovery of MTC. The National Comprehensive Cancer Network (NCCN) guidelines for breast cancer recommend against routine imaging studies or tumor marker measurements for staging asymptomatic patients with newly diagnosed stage 0 to II (early-stage) breast cancer. This is because the incidence of asymptomatic metastasis in these patients is low, and routine use of these tests is not associated with improved outcomes [9]. Instead, the NCCN guidelines recommend a comprehensive medical history and physical examination, along with an evaluation of regional lymph nodes using imaging studies such as mammography, ultrasound, or MRI. These tests can help determine the extent of the disease and guide treatment decisions [9,10].

However, tumor marker measurements such as CA15-3, CEA, and CA27-29 may be considered for monitoring response to treatment and disease progression in patients with metastatic breast cancer or in those with locally advanced or recurrent disease. In these cases, serial measurements of tumor markers can help assess the effectiveness of treatment and detect disease recurrence [10,11]. It's important to note that the use of imaging studies and tumor marker measurements should be individualized based on a patient's specific clinical presentation and guided by the treating physician in accordance with the latest guidelines and clinical evidence.

In our case, serum calcitonin was negative, and immunohistochemical staining of the excised tissue also showed negative results for calcitonin. In such cases, positivity for TFT-1, a transcription factor regulating the expression of thyroid-specific genes, could be highly suggestive of a thyroid tumor, which is often detected in both MTC and calcitonin-negative MTC [12]. Thyroidectomy for early-stage MTC may have saved the patient's life in this case by preventing metastasis of the tumor. The British Thyroid Association guidelines recommend that all patients with MTC >5 mm should undergo total thyroidectomy and level VI dissection, with further neck dissection as indicated by nodal involvement. RET-positive family members should also be offered prophylactic thyroidectomy [13]. Patients with MTC who have no evidence of lymph node metastasis and are treated early in the disease have a good prognosis with a low risk of recurrence, as seen in this case.

## Conclusions

In summary, this case highlights the significance of monitoring serum CEA levels as a diagnostic biomarker for MTC, especially in cases where serum calcitonin is negative. The absence of commonly elevated biomarkers in cancer surveillance presents a challenge for diagnostics, and only a few studies have shown MTC patients with elevated CEA levels. Early detection and treatment of MTC through thyroidectomy can prevent metastasis and improve the patient's prognosis. Lastly, it is important to note that accurate diagnosis and appropriate management of thyroid tumors require a multidisciplinary approach involving various specialists, including endocrinologists, surgeons, and pathologists.
